# Applications of Artificial Intelligence to Prostate Multiparametric MRI (mpMRI): Current and Emerging Trends

**DOI:** 10.3390/cancers12051204

**Published:** 2020-05-11

**Authors:** Michelle D. Bardis, Roozbeh Houshyar, Peter D. Chang, Alexander Ushinsky, Justin Glavis-Bloom, Chantal Chahine, Thanh-Lan Bui, Mark Rupasinghe, Christopher G. Filippi, Daniel S. Chow

**Affiliations:** 1 Department of Radiology, University of California, Irvine, Orange, CA 92868-3201, USA; rhoushya@hs.uci.edu (R.H.); changp6@hs.uci.edu (P.D.C.); jglavisb@hs.uci.edu (J.G.-B.); cchahin1@hs.uci.edu (C.C.); thanhltb@hs.uci.edu (T.-L.B.); mrupasin@hs.uci.edu (M.R.); chowd3@hs.uci.edu (D.S.C.); 2 Mallinckrodt Institute of Radiology, Washington University Saint Louis, St. Louis, MO 63110, USA; aushinsky@wustl.edu; 3 Department of Radiology, North Shore University Hospital, Manhasset, NY 11030, USA; sairaallapeikko@gmail.com

**Keywords:** prostate carcinoma, prostate mpMRI, machine learning, artificial intelligence, deep learning, neural network

## Abstract

Prostate carcinoma is one of the most prevalent cancers worldwide. Multiparametric magnetic resonance imaging (mpMRI) is a non-invasive tool that can improve prostate lesion detection, classification, and volume quantification. Machine learning (ML), a branch of artificial intelligence, can rapidly and accurately analyze mpMRI images. ML could provide better standardization and consistency in identifying prostate lesions and enhance prostate carcinoma management. This review summarizes ML applications to prostate mpMRI and focuses on prostate organ segmentation, lesion detection and segmentation, and lesion characterization. A literature search was conducted to find studies that have applied ML methods to prostate mpMRI. To date, prostate organ segmentation and volume approximation have been well executed using various ML techniques. Prostate lesion detection and segmentation are much more challenging tasks for ML and were attempted in several studies. They largely remain unsolved problems due to data scarcity and the limitations of current ML algorithms. By contrast, prostate lesion characterization has been successfully completed in several studies because of better data availability. Overall, ML is well situated to become a tool that enhances radiologists’ accuracy and speed.

## 1. Introduction

Prostate carcinoma (PCa) is the most common cancer and the third leading cause of cancer-related death among men in the United States [1]. A major challenge for PCa management is the lack of non-invasive tools that can differentiate aggressive versus non-aggressive cancer types [2]. This limitation can result in overdiagnosis and overtreatment, as evidenced by the fact that only one death is prevented for every 48 patients treated for PCa [3]. This overdiagnosis and overtreatment can lead to unnecessary biopsies, surgeries, radiotherapy, chemotherapy, and patient anxiety [2]. Better diagnostic methods could mitigate these unwarranted procedures. To meet this need for more effective screening, multiparametric magnetic resonance imaging (mpMRI) could be implemented to examine the entire prostate.

MpMRI of the prostate has been increasingly used for PCa screening in recent years [4]. MpMRI’s current utility in screening stems from a high negative predictive value for prostate cancer. However, the full potential of mpMRI has not yet been achieved [5]. PCa overdiagnosis could be reduced with an mpMRI analysis that accomplishes better lesion detection, lesion classification (benign versus malignant), and lesion volume quantification. 

Accurate prostate segmentation and volume estimation can provide invaluable information for the diagnosis and clinical management of benign prostatic hyperplasia (BPH) and PCa. This can improve BPH treatment, surgical planning, and predictions of PCa prognosis [6,7,8]. Prostate segmentation is necessary for magnetic resonance imaging (MRI) transrectal ultrasound (TRUS) fusion biopsy, which is increasingly used to diagnose PCa. MRI/TRUS fusion biopsy yield depends on accurate prostate segmentation on magnetic resonance images because the prostate edges form the reference frames for fusion with the ultrasound data [8]. Therefore, any inaccuracy in tracing prostate boundaries may lead to biopsy errors [9]. In addition to segmentation, prostate volume estimation is also a useful metric, especially with regard to BPH treatment, surgical planning, and PCa prognosis. BPH is one of the most common diseases that affects elderly men and reaches a prevalence of 90% by the ninth decade of life [10]. Large prostate volumes in men with BPH indicate a higher likelihood of more severe lower urinary tract symptoms and urinary retention [11,12,13]. Furthermore, studies have shown that patients have differential responses to BPH-targeted medications, depending on prostate size [11]. Additionally, prostate volume is considered when determining a surgical approach, with each procedure having its own risk profile [14]. In addition to guiding BPH treatment, prostate volume is used in PCa prognosis. Prostate size alone is a valuable marker for PCa prognosis; PCa is more accurately detected in prostates under 50 cm^3^ than in those over 50 cm^3^ [6]. Prostate volume is also used to calculate prostate-specific antigen density, a figure that helps to differentiate BPH from PCa and can also be used to predict radical prostatectomy outcomes [15,16,17].

Accuracy of prostate lesion detection, segmentation, and volume estimation is important at different stages of PCa management. Lesion detection identifies regions for biopsy. Accurate segmentation is crucial for improved fusion biopsy yields. Additionally, segmentation improves radiotherapy delivery. Volume estimation predicts prognosis after prostatectomy [18,19,20]. Prostate lesion detection is crucial because the effective treatment of PCa directly depends on identifying cancer at its earliest stage [21,22,23]. Even though PCa most often follows an indolent course, it can show rapid progression in some cases. In these instances, lesion recognition on mpMRI is critical because it provides a region of high suspicion and a higher yield from targeted biopsy [24]. Without mpMRI lesion detection, random 12 core TRUS biopsies are performed, which may miss small or anteriorly located PCa [25].

While early prostate lesion detection improves timely PCa treatment, accurate lesion segmentation can improve radiotherapy [26]. Prostate lesion contouring is a major source of error when administering radiation therapy. This inexact segmentation can lead to the underdosage of the tumor as well as the overdosage of normal cells [20]. Although radiotherapy is an effective cancer treatment, its use is hampered by imprecise delineation. More precise contouring of a malignant lesion can improve lesion targeting and relative radiotherapeutic dosage, which can lead to lower recurrence rates [26,27].

Pre-operative prostate lesion volume estimation is a key metric for predicting the likelihood of positive surgical margins, biochemical prostate-specific antigen (PSA) recurrence, and cancer-specific survival post-prostatectomy [28,29,30,31]. This volume is a better indicator of surgical margins than other factors such as Gleason score and extracapsular extension [28]. Lesion volume also functions as an independent variable for PSA recurrence, an early sign of recurrent disease which may require salvage radiation therapy [32]. In addition, lesion volume predicts cancer-specific survival more accurately than variables such as lymphadenopathy, seminal vesical invasion, and Gleason score [29].

After prostate lesions have been detected on mpMRI, lesion characterization is important for selecting appropriate management options. Accurate prostate lesion classification on mpMRI could preclude biopsies in men with low-grade tumors, reduce the number of biopsy cores, and decrease the rate of overdiagnosis and false-negative biopsies [33]. Reduction in unnecessary biopsies is important, as potential TRUS biopsy complications include hematuria, lower urinary tract symptoms, and temporary erectile dysfunction [34]. Additionally, the number of biopsy cores obtained correlates with increased risk of complications, including rectal bleeding, hematospermia, bleeding complications, and acute urinary retention [34]. Furthermore, the overdetection of PCa exerts a major psychological toll on quality of life and increases the risk of overtreatment [2]. Overtreatment side effects that may occur after radical prostatectomy and radiotherapy include urinary incontinence, rectal bleeding and fistulae, and erectile dysfunction [2,35,36,37].

Artificial intelligence (AI) is a promising tool to improve prostate lesion detection, lesion characterization, and lesion volume quantification. AI can systematically evaluate mpMRI images [38]. Machine learning (ML), a branch of AI, and its sub-discipline, deep learning (DL), have become attractive techniques in medical imaging because of their ability to interpret large amounts of data [39]. By applying ML to prostate mpMRI data, imaging-based clinical decisions could be improved. The purpose of this review is to summarize ML applications for prostate mpMRI in regards to (1) prostate organ segmentation, (2) prostate lesion detection and segmentation, and (3) prostate lesion characterization.

## 2. Multiparametric Magnetic Resonance Imaging

Multiparametric magnetic resonance imaging (mpMRI) of the prostate is a form of advanced non-invasive imaging that combines standard anatomical sequences with functional imaging. It consists of T1-weighted images, T2-weighted images (T2W), and the following functional sequences: diffusion-weighted images (DWI) including the apparent diffusion coefficient maps (ADC) and dynamic contrast-enhanced images (DCE). Certain protocols also incorporate proton magnetic resonance spectroscopy imaging (MRSI) [40,41]. Typically, the functional techniques used are DWI and DCE. MRSI is more demanding than DWI and DCE because it requires more acquisition time, greater technical expertise, and intensive post-processing of the data. Therefore, it is not commonly used [42]. 

The advantages of seeing both the anatomy and functional ability of the prostate have made mpMRI an attractive imaging technique with many applications. It can accurately identify clinically relevant cancer. The combination of T2W, DWI, and DCE has high specificity, sensitivity, and negative predictive value in detecting PCa [43,44,45]. The use of all three functional sequences has been found to have a positive predictive value for PCa of 98% [46]. In addition to diagnosing PCa, mpMRI is also used in the management of the disease as the functional sequences aid in predicting tumor behavior. Prostate mpMRI has been used for active surveillance, tumor localization, staging, treatment planning, and monitoring of recurrence [40,41].

While mpMRI is a powerful imaging modality, it does have limitations. Differences in image acquisition techniques and protocols across institutions lead to heterogeneity in imaging quality and make it challenging to compare images [47]. Additionally, the learning curve for reading mpMRIs is steep, and there exists inter-observer variability [48,49,50]. The experience of radiologists reading these scans impacts the utility of prostate mpMRI images. In addition, the prostate gland is difficult to delineate, and various benign and pre-malignant processes can mimic PCa [51]. For example, the sensitivity for the detection of PCa in the transitional zone is limited by the heterogeneous nature of this zone in the setting of BPH, which can also exhibit increased cellularity further complicating the distinction. Furthermore, patient-related factors, including body habitus, prior procedures, and unconventional anatomy, can impact imaging. Artifacts, such as field inhomogeneity from rectal gas and metal implants, can substantially impede the interpretation and reporting of prostate mpMRI. Finally, it can be difficult to discriminate between post-treatment changes and local recurrence following treatment on mpMRI.

In an effort to assist in standardizing the acquisition, interpretation, and reporting of prostate mpMRI, the Prostate Imaging Reporting and Data System (PI-RADS) was developed by the European Society of Urogenital Radiology (ESUR) in 2012 [52]. The ESUR, in collaboration with the American College of Radiology and the AdMeTech Foundation, released updated versions PI-RADS v2 in 2015 and PI-RADS v2.1 in 2019 [53,54]. All of the PI-RADS versions offer guidance for protocols and specifications for image acquisition. The scoring systems provide frameworks to evaluate individual sequences of T2W, DWI, and DCE and to integrate these findings into an overall risk assessment category from 1 to 5. These risk categories assist in the determination of biopsies and the management of clinically significant PCa.

## 3. Artificial Intelligence Paradigms: Machine Learning and Deep Learning

**Figure 1:**
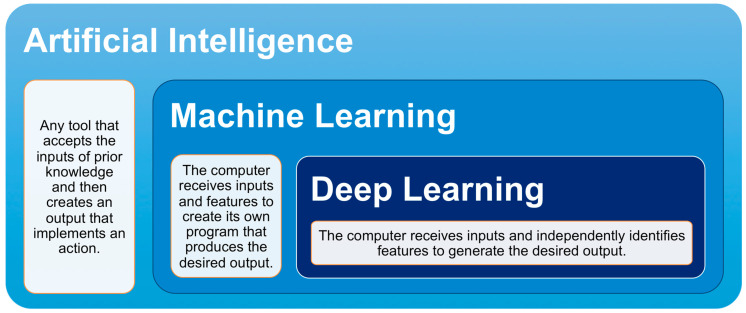
Relationship between artificial intelligence, machine learning, and deep learning. Artificial intelligence is an umbrella term that includes machine learning and deep learning. Deep learning is a hyponym of machine learning.

Although the terms AI, ML, and DL are commonly used interchangeably, each term has its own specific definition. AI is the broad, umbrella term that encompasses both ML and DL, with DL being a subset of ML (Figure 1). Marvin Minsky, an early AI developer, described AI as “the science of making machines do things that would require intelligence if done by men” [55]. AI is the ability of any tool to accept inputs of prior knowledge, experience, goals, and observations and then create an output that implements an action. This definition covers a wide range of tools varying from a simple thermostat to a self-driving car. AI research often falls under the domain of computer science because AI tools perform many computations to create appropriate outputs [56].

Whereas AI typically entails a fixed, rules-based computational method, ML dynamically improves upon computational methods as data is input and trained. In traditional programming, a computer receives data and a program as inputs and then produces the output in a one-to-one manner. All improvements to the results derive from alterations to the program rules. In ML, a computer receives data and labels as inputs and then creates a program to refine the outputs. The computer learns by comparing its own outputs, also known as predictions, to data that has already been defined and associated with a label. Over time, the ML algorithm will improve upon its ability to create a program that can match its own output to a label. The effectiveness of the program is highly dependent on the quality and size of data that the ML algorithm receives as input. 

The data types that can be input into an ML algorithm vary widely, encompassing digitized handwriting, text from documents, DNA sequences, facial images, and more. A ML algorithm can utilize this data to train and make predictions. Two of the most common ML implementations are classification and regression [57]. In classification, ML receives data and then decides upon a category for each item in the data. For example, ML could look at images and decide whether the image is a plane, car, or boat. In regression, ML receives data and then predicts a numerical value for each item in the data. Examples include predicting tomorrow’s ambient temperature or the price of a stock.

**Figure 2:**
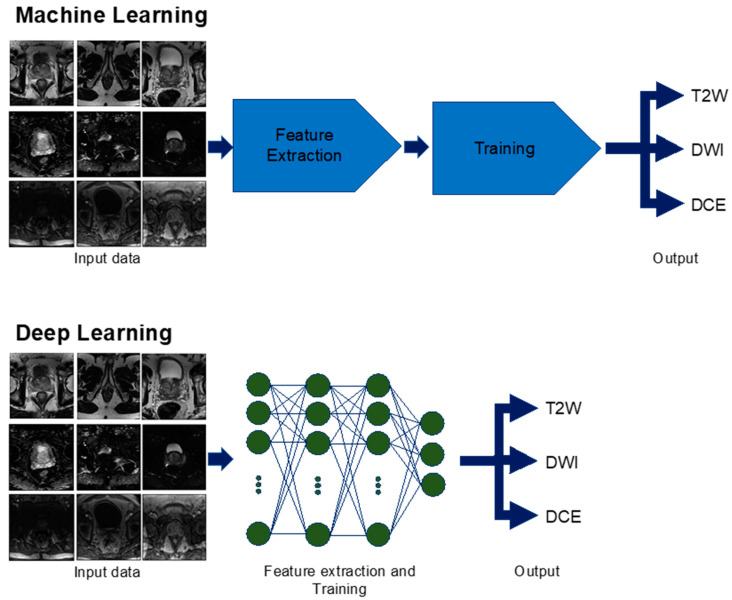
Machine learning versus deep learning used for multiparametric magnetic resonance imaging (mpMRI) sequence identification. In machine learning, the computer receives inputs of mpMRI images and goes through feature extraction specific to the different sequences of T2-weighted (T2W), diffusion-weighted imaging (DWI), and dynamic contrast-enhanced (DCE). Then, the computer is trained on additional images and is able to identify the correct sequence as an output. Deep learning differs from machine learning in that feature extraction and training can be done simultaneously to produce the output.

Within the ML discipline, DL has garnered significant attention because of the groundbreaking results that it achieved in the ImageNet Large Scale Visual Recognition Challenge competition, where competitors developed algorithms using a subset of a public dataset of images [58]. DL has flourished with the rise of big data and faster hardware [39]. In traditional ML, the algorithm has features that it will extract from the data before training begins [57] (Figure 2). These features are constant and are based upon established rules. For example, the algorithm can look for eyes when trying to recognize a face or search for wings when identifying an airplane. By contrast, a DL algorithm does not require feature selection before training. DL simply receives input and learns its salient features during training (Figure 2). DL architecture is also notable because it is formed by many tiered layers, which resemble a brain’s neuronal network. These layers enable DL to extract features from progressively smaller sizes of input data and allow for increased feature complexity [59]. Although various DL architectures exist, convolutional neural networks (CNN) are considered well suited for medical imaging. The overall goal of these techniques is to allow the machine to determine and optimize features automatically for evaluating and classifying images. 

Medical imaging studies that use ML algorithms are frequently designed with three dataset types: training, validation, and test [60]. The study will first use training data as its input to develop an algorithm that produces the desired output. During this training period, the algorithm constantly uses validation data to provide correct feedback to modify itself. After the algorithm has finished development, final performance is then assessed with test data. Because test data was not used during the algorithm training, it is an objective method to assess performance.

## 4. Prostate Organ: Segmentation and Volume Estimation

Although prostate segmentation and volume approximation could greatly improve PCa and BPH management, existing techniques are limited. Currently, prostate segmentation is performed in a manual or semi-automated fashion and is limited by inter-observer variability [61]. According to a study by Rash et al. [62], the mean prostate organ volume among three radiation oncologists varied between 0.95 and 1.08. Currently, prostate volume is most often calculated during TRUS utilizing an ellipsoid estimate [63] or estimated during a prostate exam. Even though this volume approximation with TRUS is commonly used, it has significant intra-observer variation and is not as accurate as an approximation with mpMRI images [64,65]. Prostate volume approximation with software has been attempted with limited results. Medical students outperform the accuracy of a commercially available tool [66]. 

**Figure 3:**
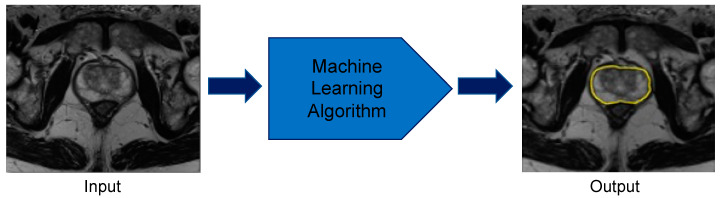
Prostate organ segmentation performed by machine learning methods. The computer takes multiparametric magnetic resonance imaging images as inputs and applies the developed machine learning algorithm to correctly identify the borders of the prostate.

To meet this need for an automatic, accurate prostate segmentation and volume approximation tool, ML methods have been applied by various groups (Figure 3). A ML technique, fuzzy c-means clustering, categorizes data into groups via unsupervised learning and was used by Rundo et al. [67] to segment the prostate on T1-weighted and T2-weighted mpMRI images. Rundo et al. evaluated 21 patients to yield an average Dice score of 0.91 [67]. The Dice score is a standard statistic for assessing the spatial intersection between two images and ranges from 0 (no overlap) to 1 (perfect overlap) [68]. Therefore, a Dice score of 0.91 demonstrates that the technique was able to segment and estimate the volume of prostates with a high level of precision. 

Besides fuzzy c-means clustering, DL has been extensively used for complete prostate segmentation. In 2012, the release of the PROMISE12 challenge dataset, which contained 100 patients, prompted many studies on this topic [69,70]. Two groups led by Tian et al. [71] and Karimi et al. [70] both employed CNNs. Tian et al. [71] trained their CNN on T2-weighted mpMRI images from 140 patients and achieved a Dice score of 0.85. Karimi et al.’s [70] CNN was trained on a limited dataset of 49 T2-weighted mpMRI images supplemented by data augmentation. Their Dice score was 0.88. Both studies achieved high Dice scores and demonstrated that prostate segmentation could be achieved with commonly used technical designs.

Additionally, a uniquely designed DL network for biomedical images, U-Net, has also been proposed for complete prostate segmentation [72]. U-Net is an algorithm that successively compresses an image, derives features during these contractions, and classifies every pixel in the image [72]. Three studies used U-Net for prostate segmentation and obtained Dice scores of 0.89, 0.93, and 0.89 [73,74,75]. These three groups showed that U-Net could effectively segment the prostate with dataset sizes between 81 and 163 patients. The high Dice scores across multiple studies with comparable network architectures demonstrate substantial progress towards completely automated prostate segmentation and volume approximation. Table 1 lists the previously discussed studies along with several others that also segmented the prostate using various CNNs. To establish the ground truth label, which is used in establishing a Dice score, five studies used radiologists, two studies used clinicians of unstated specialties, one study used an expert, and one study used a radiologist for most of its data and an unnamed source for the rest of its data [67,70,71,73,74,75,76,77,78].

## 5. Prostate Lesion: Detection, Segmentation, and Volume Estimation

Although prostate lesion detection, segmentation, and volume approximation could benefit PCa management, an effective tool that can automate these processes has not been created. For prostate lesion detection, satellite small lesions can be challenging to detect [19]. In a study by Steenbergen et al. [19], six different teams, each composed of one radiologist and one radiation oncologist, missed 66 out of 69 satellite lesions distributed across 20 patients. In addition to prostate lesion detection, segmentation is difficult because sparse tumors composed of benign glands and stroma are challenging to outline [79]. When segmentation across multiple institutions is compared, the contours reveal considerable differences [80]. As a result of inexact segmentation, volume approximation of prostate lesions is also challenging and often underestimates the histopathological volume [79]. This need for improved lesion metrics could be satisfied using ML algorithms that could learn to identify these features within mpMRI images.

**Figure 4:**
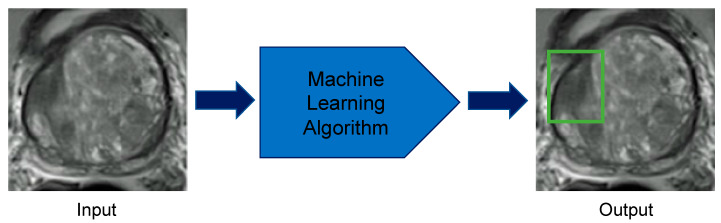
Prostate lesion detection using machine learning methods. The computer takes multiparametric magnetic resonance imaging images of the prostate as inputs and applies the developed machine learning algorithm to correctly localize lesions in the prostate.

For prostate lesion detection, ML approaches have been used to identify potential malignancies (Figure 4). Lay et al. [81] used a prostate computer-aided diagnosis (CAD) based on a random forest for prostate lesion detection (Table 2). This study’s dataset used 224 patient cases across three sequences (T2-weighted, ADC, and DWI) for a total of 287 benign lesions and 123 lesions with a Gleason score of 6 or higher [81]. The Gleason scoring system describes PCa grades on a scale of 1 to 5 based on the pattern that the cancerous cells fall into, with 1 or 2 being low grade and 5 being high grade. It uses the combined grades of the most prominent and second most prominent patterns in a biopsy as the final score. A Gleason score of 6 or greater has malignant potential [82]. Lay et al.’s random forest technique yielded an area under the curve (AUC) score of 0.93 [81]; AUC is a measurement for binary classification and ranges from 0 to 1. Therefore, this study demonstrates that the ML model can detect lesions with high accuracy.

DL techniques have also been applied to prostate lesion detection (Table 2). Xu et al. [84] implemented a type of neural network with extensive layers, ResNet [86], to find lesions on T2-weighted, ADC, and DWI images. This study used images from the Cancer Imaging Archive data portal and included 346 patients. They achieved an AUC of 0.97 [84]. Tsehay et al. [85] also used a DL algorithm with a 5-layer CNN architecture that used an individual loss function for each layer. The CNN was trained and validated on a dataset of 39 benign lesions and 86 lesions with a Gleason 6 or higher [85]. Tsehay’s group achieved an impressive AUC of 0.90 [85], which demonstrates high accuracy of prostate lesion detection. All four studies in Table 2 used radiologists for labeling the ground truth [81,83,84,85]. 

**Figure 5:**
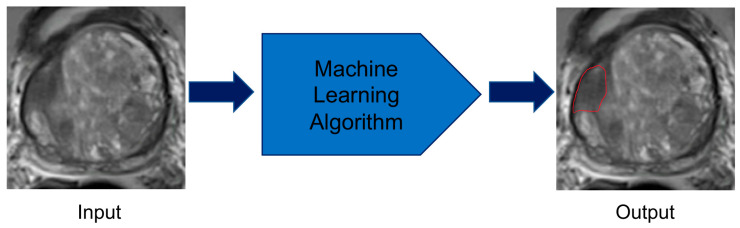
Prostate lesion segmentation using machine learning techniques. The computer takes multiparametric magnetic resonance imaging images of the prostate as inputs and applies the developed machine learning algorithm to correctly identify the borders of the lesion.

Although prostate lesion detection has been implemented with ML, automated prostate lesion segmentation and volume approximation remain largely unsolved (Figure 5). Few studies have attempted this task due to a dearth of well-curated data and its technical requirements. One obstacle for prostate lesion segmentation is a lack of guidelines across institutions for prostate lesion contours, which results in significant inter-observer variability [19,80]. Despite the lack of standardization, three studies have attempted prostate lesion segmentation (Table 3). A study by Liu et al. [87] used fuzzy Markov random fields to achieve a Dice score of 0.62 with 11 patients. Two other groups, Kohl et al. [88] and Dai et al. [89], both employed DL algorithms and used U-Net and Mask R-CNN, respectively. Kohl’s group used a dataset of 152 patients and implemented U-Net combined with an adversarial network. Their architecture resulted in an average Dice score for prostate lesion segmentation of 0.41 [88]. Dai’s group used a highly specialized DL algorithm, Mask R-CNN, and trained with 63 patients to achieve a prostate lesion Dice score of 0.46 [89]. To label the ground truth, Dai et al. [89] used a clinician, Kohl et al. [88] used a radiologist, and Liu et al. [87] used a pathologist. These studies’ lower Dice scores demonstrate that the current techniques have limited precision. These studies show that prostate lesion segmentation and volume estimation remain challenging. A bigger dataset with more uniform labeling would permit the development of more ML models geared toward these tasks.

## 6. Prostate Lesion: Characterization

Although prostate lesions have been increasingly imaged with mpMRI since 2013 [4], their characterization has been hindered by the variability in classification conventions across different radiologists and institutions [4,47,90]. To establish better standardization, the PI-RADS scoring system was created in 2012, with an updated version PI-RADS v2 released in 2015, and the newest version PI-RADS v2.1 released in 2019 [53,54,91]. Since their conception, multiple studies have attempted to elucidate the clinical utility of PI-RADS, PI-RADS v2, and PI-RADS v2.1. Challenges to its broader acceptance include inter-reader agreement, radiologist experience, and the substantial interpretation time of images [4,47,90]. This need for more consistent lesion characterization makes ML an attractive method for accurate, quick classification. 

ML algorithms can augment the PI-RADS scoring system as well as independently classify lesions (Table 4). Regarding PI-RADS, Litjens et al. [92] created a CAD system that applied a random forest for characterizing prostate lesions on a scale of suspicion for malignancy. After combining the ML generated scores and the radiologist provided PI-RADS scores on a dataset of 107 patients, the overall AUC was greater than either the ML generated scores or the PI-RADS scores [92]. Similarly, Wang, J. et al. [93], who used 54 patients in their dataset, also concluded that a support vector machine (SVM) algorithm enhanced the PI-RADS performance of radiologists. Song et al. [94] opted to use a DL algorithm based off of VGG-Net, a deep CNN, as a tool for improving PI-RADS scores assigned by radiologists. Song’s group gathered data from 195 patients and also observed that their AUC improved when radiologists’ decisions were combined with the VGG-Net [94].

**Figure 6:**
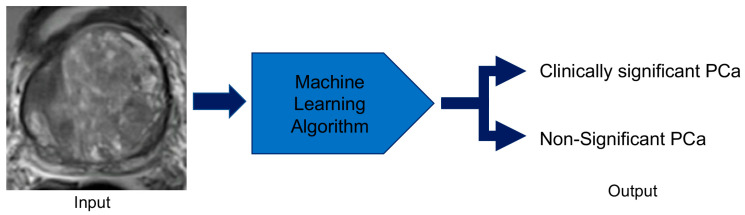
Prostate lesion characterization using machine-learning techniques. The computer receives multiparametric magnetic imaging images of prostate lesions and applies the developed machine learning algorithm to categorize the lesion as clinically significant prostate cancer or non-significant prostate cancer.

In addition to bolstering lesion classification by radiologists, ML algorithms have been trained to characterize prostate lesions independently (Figure 6, Table 4). Many studies explored this task with the PROSTATEx challenge dataset that was released in 2017 [101]. The PROSTATEx dataset was gathered from 344 patients and contained segmented lesions along with their respective pathology-defined Gleason scores [101]. From this public database, Wang, Z. et al. [96] achieved an AUC of 0.96 by running two CNNs in parallel. Both Seah et al. [97] and Liu et al. [98] obtained an AUC of 0.84 by using deep layered CNNs. Mehrtash et al. [99] implemented a 3D CNN to reach an AUC of 0.80. One study by Kwak et al. [95] used its own proprietary dataset to implement an SVM that trained on T2-weighted and DWI images to characterize prostate lesions. In this study, 244 patients were used for a total of 333 benign and 146 malignant lesions [95]. The SVM method used discriminative features in training that resulted in an AUC score of 0.89 [95]. All of the studies listed in Table 4 used radiologists to determine their ground truth [77,92,93,94,95,97,98,99,100]. These studies highlight the ability of DL algorithms to predict the likelihood of a lesion’s malignancy based upon Gleason scores. 

## 7. Future Work

The potential applications of ML to PCa surpass volume estimation, lesion detection, and lesion characterization. Further developments in prostate lesion classification may lead to a more practical clinical use, include training ML algorithms for tumor grade prediction. In addition to analyzing data solely from images, ML could augment the clinical management of PCa by incorporating demographic and biochemical data. ML could enable clinicians to make more assured decisions regarding the need for biopsy, medication dosing, and cancer recurrence. Biopsies that are performed for diagnosing PCa could be rendered unnecessary with a ML tool. Two studies by Hu et al. [102] and Chen et al. [103] used data such as age, digital rectal exam findings, PSA, and prostate volume for biopsy prediction. These studies made accurate PCa diagnoses and showed the potential for ML to eliminate the need for biopsy. In addition to diagnosis, ML could impact PCa medication dosing in PCa management. Radiation therapy requires accurate dosing, which is frequently operator dependent [104]. By minimizing operator dependency, ML could offer better standardization leading to more precise dosing. Nicola et al. [105] employed ML to predict prostate brachytherapy dosing by analyzing images and prior treatment plans from other patients. This study showed that ML implementation was comparable to brachytherapists and could be advanced by using a DL instead of a traditional ML algorithm. Along with diagnosis and dosing, ML could be used for predicting cancer recurrence after prostatectomy. Two studies by Wong et al. [106] and Cordon et al. [107] gathered data such as Gleason score, PSA, seminal vesical invasion, and surgical margins to predict recurrence after prostatectomy. The accuracy of these studies could be increased by adding postoperative imaging data for improved recurrence prediction.

## 8. Conclusions

AI applications in prostate mpMRI are promising tools for more effective and efficient image interpretation, leading to improved care. In pure image interpretation, ML has shown noteworthy progress in prostate organ segmentation and volume estimation. As better-curated data becomes available for prostate lesions, ML will likely become more successful at lesion detection, volume estimation, and characterization. As ML evolves, it will indisputably change radiologists’ workflow by performing many of the simple tasks in image interpretation. However, ML will not replace the role of radiologists, who are critical to solving complex clinical problems [104]. AI is poised to enhance the decisions made by radiologists. It will enable radiologists to better care for their patients rather than supersede the need for radiologists. 

Similarly, ML’s ability to evaluate complex datasets across different domains suggests this technique may facilitate the bridging of advanced imaging, such as mpMRI, with emerging biomarker analysis or tumor genetics. Thus, ML may form the underpinnings of radiogenomics, allowing for the integration of imaging data, blood chemistry analysis, and pathologic evaluation in forming complex models that can predict treatment response. Enabled by larger datasets and more sophisticated mathematical techniques, ML could progress to creating completely automated tools that receive a patient’s prostate mpMRI images and then delineate a range of desired features, as well as giving likelihood metrics for an array of pathologies. 

## Figures and Tables

**Table 1:** Machine learning techniques applied to prostate organ segmentation.

Reference	Year	ML Algorithm	Patients	Dice	Modalities
Rundo et al. [67]	2017	Fuzzy C-means clustering. Features: T1 intensity, T2 intensity	21	0.91	T1W, T2W
Tian et al. [71]	2018	CNN: 7 layers	140	0.85	T2W
Karimi et al. [70]	2018	CNN: 3 layers	49	0.88	T2W
Clark et al. [73]	2017	CNN: U-Net	134	0.89	DWI
Zhu, Y. et al. [74]	2018	CNN: U-Net	163	0.93	DWI, T2W
Zhu, Q. et al. [75]	2017	CNN: U-Net	81	0.89	T2W
Milletari et al. [76]	2016	CNN: V-Net	80	0.87	T2W
Wang, B. et al. [77]	2019	CNN: 3D DSD-FCN	40	0.86	T2W
Cheng et al. [78]	2016	CNN and Active Appearance Model	120	0.93	T2W

**Table 2:** Machine learning techniques applied to prostate lesion detection.

Reference	Year	ML Algorithm	Patients	Lesions	AUC	Modalities
Lay et al. [81]	2017	Random Forest. Features: Intensity, Haralick texture	224	410	0.93	T2W, ADC, DWI
Sumathipala et al. [83]	2018	CNN: Holistically Nested Edge Detection	186	N/A	0.93	T2W, ADC, DWI
Xu et al. [84]	2019	CNN: ResNet	346	N/A	0.97	T2W, ADC, DWI
Tsehay et al. [85]	2017	CNN, 5 Layers	52	125	0.90	T2W, ADC, DWI

**Table 3:** Machine learning techniques applied to prostate lesion segmentation.

Reference	Year	ML Algorithm	Patients	Dice	Modalities
Dai et al. [89]	2019	CNN: Mask R-CNN	63	0.46	T2W, ADC
Kohl et al. [88]	2017	Adversarial Network and CNN: U-Net	152	0.41	T2W, ADC, DWI
Liu et al. [87]	2009	Fuzzy Markov Random Fields	11	0.62	T2W, quantitative T2, DWI, DCE

**Table 4:** Machine-learning techniques applied to prostate lesion characterization.

Reference	Year	Algorithm	Patients	Lesions	AUC	Modalities
Litjens et al. [92]	2015	Random Forest. Features: Intensity, Position, Pharmacokinetic, Texture, Spatial Filter	107	141	Benign vs. Cancer; AUC increased from 0.81 to 0.88 with their ML tool Indolent vs. Aggressive; AUC increased from 0.78 to 0.88 with their ML tool	T2W, DCE, DWI
Wang, J. et al. [93]	2017	SVM. Features: Volumetric Radiomics	54	149	0.95	T2W, DWI
Song et al. [94]	2018	CNN: Deep CNN and Augmentation	195	547	0.94	T2W, ADC, DWI
Kwak et al. [95]	2015	SVM. Features: Texture	244	479	0.89	T2W, DWI
Wang, Z. et al. [96]	2018	CNN: Deep CNN	360	600	0.96	T2W, ADC
Seah et al. [97]	2017	CNN: Deep CNN	346	538	0.84	T2W, ADC, DCE
Liu et al. [98]	2017	CNN: XmasNet	341	538	0.84	T2W, ADC, DWI, Ktrans
Mehrtash et al. [99]	2017	CNN: 3D Implementation	344	538	0.80	ADC, DWI, DCE
Chen et al. [100]	2019	Two CNNs: Inception V3 and VGG-16	Training Data: 204 Test Data: N/A	538	Inception V3, 0.81 VGG-16, 0.83	T2W, DWI, DCE
